# Updates in Type 1 Diabetes (T1D): What Recent Findings Might Mean for Treatment and Prevention Strategies

**DOI:** 10.1111/1753-0407.70189

**Published:** 2026-01-20

**Authors:** Zachary Bloomgarden

**Affiliations:** ^1^ Department of Medicine, Division of Endocrinology, Diabetes and Bone Disease Icahn School of Medicine at Mount Sinai New York New York USA

Recent articles show the rising population burden of T1D, illustrate current disease‐modifying/prevention efforts, and show the growing recognition of interrelationships between T1D and T2D and their implications for complications.

Epidemiologic trends: There has been a considerable rise in T1D prevalence. Analysis using the Global Burden of Disease Study dataset estimated that there were 2.95 million people with T1D age 40–64 in 1990, increasing to 7.4 million in 2021, an increase in prevalence from 274 to 346 per 100 000 population, with predicted further increase to 437 per 100 000 in 2036, with part of the increase in prevalence related to the decrease in mortality from 1.16 to 0.82 per 100 000 during this period, a level projected to be stable over the coming decade [1]. T1D prevalence is greater with increasing age and, even more, with higher sociodemographic index [1].

Interventions to delay or prevent T1D: With more people living longer with T1D, and more individuals identified earlier in the disease course, the practical question becomes which interventions can meaningfully delay progression and reduce future burden. An interesting “real world” study addressed the use of teplizumab, a monoclonal antibody directed at T cell antigen recognition and indicated to delay the onset of clinically symptomatic (Stage 3) T1D in antibody‐positive patients with abnormal glucose levels that have not caused clinical symptoms (Stage 2). The TriNetX dataset of > 150 million people was queried, finding 42 who had been treated with teplizumab; 83% of them did not require insulin at mean 197‐day followup [2], somewhat less than the 93% at 12 months in a meta analysis of randomized controlled trials (RCT) of this agent, who showed decreased insulin requirement through 24 months, although without significant reduction in HbA1c beyond 12 months [3]. As teplizumab was approved by the FDA in November 2022, it should soon be feasible to obtain information about 3‐ and 4‐year effects of this agent in clinical practice to better understand its potential benefit.

Immunosuppressive therapy is one path to delaying clinical disease; another is antigen‐specific prevention, intended to reduce risk without broad immunosuppression. Administration of oral insulin to relatives of people with T1D is attractive as a non‐immunosuppressive approach. Studies have not, however, revealed overall delay in progression to stage 3 T1D with oral insulin [4], although clinical trials have shown variation between participants, with oral insulin decreasing progression among those having low first‐phase insulin release [5]. To further analyze this, genotyping of 552 participants in the TrialNet Oral Insulin Prevention Trial TN07 study found that a higher T1D genetic score, positive insulin autoantibodies, and lower proinsulin and obesity‐related polygenic scores (which would suggest characteristics of T2D) might be related to progression of at‐risk relatives to T1D and might help in selection of candidates for this approach [6].

If prevention effects vary across subgroups, the next step is to better map the underlying heterogeneity among autoantibody‐positive individuals, both genetically and phenotypically. In a study of 4324 islet autoantibody‐positive TrialNet Pathway to Prevention participants without clinical diabetes and with a median age of 11.3 years, those with higher insulin secretion based on C‐peptide response to oral glucose as well as higher glucose response had a greater type 2 diabetes (T2D) genetic risk score, while those with lower insulin secretion and higher glucose had a greater T1D genetic risk score [7]. Those with the highest C‐peptide were older, were more often only one autoantibody positive, had a higher BMI Z‐score, and had greater insulin resistance based on HOMA‐IR score (the product of fasting insulin and fasting glucose); the effect of T2D genetic risk score on the likelihood of developing stage 3 T1D was less pronounced than that of the T1D genetic risk score [2].

Interrelationships betweeen T1D and T2D: Epidemiologic observations point in the same direction, suggesting that T2D risk factors can meaningfully interact with T1D development. Earlier studies have shown that a positive family history of T2D may increase the risk of offspring developing T1D [8]. Similarly, the characteristics of patients with T1D having family members with T2D, having older age at onset, higher body weight, higher levels of endogenous C‐peptide, and lower presence of islet autoantibodies and T1D‐susceptibility HLA genotypes have been observed in other populations with T1D [9, 10]. An additional important consideration is that higher T2D genetic risk may lead people developing T1D to have greater future risk of complications. In the EURODIAB study, waist‐hip ratio, low HDL cholesterol, and high triglyceride were risk factors for coronary heart disease [11]. Similarly, in the DCCT/EDIC study, characteristics of metabolic syndrome were associated with higher coronary artery calcium and carotid intima‐media thickness [12].

This overlap between T1D and T2D is also evident clinically in the roles of body weight and insulin resistance in both conditions, with obesity and insulin resistance common enough in T1D that they increasingly shape clinical management. A recent publication began by affirming that “excess weight is no longer a distinguishing feature between patients with T1D and those with T2D,” reviewing a number of potentially useful approaches to this, including avoidance of medications associated with weight gain and reviewing the evidence for approaches to weight loss in people with T1D treated with GLP‐1RA, pramlintide, SGLT2i, and metformin, as well as addressing consideration of bariatric surgery in some people with T1D [13].

Across these observations runs a consistent message: immune and metabolic mechanisms can intersect, with their interaction having practical implications for both prevention and complications. Aspects of T1D development and complications are then importantly associated with T2D characteristics [14, 15]. T1D and T2D should no longer be considered entirely distinct conditions but rather represent different states of decreased insulin action leading to future complications, heeding Edwin Gale's thoughtful observation “that immune‐mediated processes are relevant to the pathogenesis of type 2 diabetes, and that non‐immune processes may also play a role in type 1 diabetes” [16].

## Funding

The author has nothing to report.

## Conflicts of Interest

The author declares no conflicts of interest.
